# Supplementary material for: Fluorescent sensors for imaging of interstitial calcium
Source: Nat Commun. 2023 Oct 5;14:6220. doi: 10.1038/s41467-023-41928-w (PMC10556026; doi:10.1038/s41467-023-41928-w)
Supplement: Supplementary file 1 — Supplementary Information [file 41467_2023_41928_MOESM1_ESM.pdf]

Supplementary Information

Valiente-Gabioud et al.

Fluorescent Sensors for Imaging of Interstitial Calcium

Supplementary Table 1. Crystallization of variant NRS 1.2

**Data collection and refinement statistics.**	
Wavelength	1
Resolution range	47.85 - 1.279 (1.325 - 1.279)
Space group	P 21 21 21
Unit cell	54.332 62.854 95.709 90 90 90
Total reflections	1047921 (90739)
Unique reflections	85238 (8382)
Multiplicity	12.3 (10.8)
Completeness (%)	99.97 (99.73)
Mean I/sigma(I)	15.60 (0.94)
Wilson B-factor	15.85
R-merge	0.08807 (2.069)
R-meas	0.09188 (2.171)
R-pim	0.02593 (0.6485)
CC1/2	0.999 (0.389)
CC*	1 (0.748)
Reflections used in refinement	85233 (8382)
Reflections used for R-free	4303 (405)
R-work	0.1832 (0.3259)
R-free	0.2005 (0.3231)
CC(work)	0.962 (0.671)
CC(free)	0.967 (0.672)
Number of non-hydrogen atoms	2624
macromolecules	2249
ligands	44
solvent	343
Protein residues	282
RMS(bonds)	0.01
RMS(angles)	1.11
Ramachandran favored (%)	99.64
Ramachandran allowed (%)	0.36
Ramachandran outliers (%)	0
Rotamer outliers (%)	0.41
Clashscore	7.05
Average B-factor	21.46
macromolecules	20.1
ligands	23.85
solvent	30.16

Statistics for the highest-resolution shell are shown in parentheses.

Supplementary Note. Directed Evolution of GreenT-EC

Parental protein and linker optimizations

The starting parental construct for GreenT-EC was obtained by replacing tryptophan 148 in mNeonGreen with the minimal calcium binding domain of Troponin C (TnC) previously optimized during the design of the FRET-based "Twitch" family of indicators[1]. The four amino acid long linkers flanking the minimal domain in Twitch-2B were initially also incorporated for generating our mNeonGreen-based indicator (Supplementary Fig. 1). Twitch-2B was crystalized[2], which provided further insights into the role of the linkers, which were not only limited to provide flexibility for an optimal orientation change between the bound and apo forms of the indicator, but also established key interactions with the TnC domain and the fluorescent proteins to stabilize the conformations. The parental version of mNeonGreen-TnC had a maximal fluorescence change of about 100 %. It was subsequently subjected to extensive saturation mutagenesis in both linker regions randomizing 4 amino acid positions at the time. The first notable improvement was achieved during the optimization of linker 2 (sequence change: PIYP to LTDN at position 221-224) and brought the response of purified proteins from 100% to 600-700 %, with a Kd of 127 nM and an extinction coefficient (EC) of 67 mM^{-1} cm^{-1} in the calcium bound state. The second evolutionary step was obtained using this variant to further optimize linker 1 (sequence change: GADA to LGWD at position 148-151), reaching a maximum fluorescence change of 1100 % after calcium binding. The Kd of this variant was 221 nM and the EC was 75 mM^{-1} cm^{-1}. We also evaluated the effect of linker extensions by adding 3-7 extra amino acids to the linkers but no improvements were observed.

Region 1

We finally identified a critical region which had an eminent effect on the indicator response (sequence CRS at position 225-227) within the coding region of mNeonGreen immediately after the second linker. We named this region R1 (Supplementary Fig. 1a, b). After screening this region, we selected two main next generation variants: a variant with a single mutation C225N (named NRS) and one with a triple mutation changing the amino acids CRS to RTT (named RTT). Both variants had fluorescence changes of around 6000% after binding calcium. Interestingly, the excitation and emission maxima shifted from values of 508/517 nm in mNeonGreen to 497/515 nm in NRS and 504/515 nm in RTT. In addition, while the Kd of both variants remained in the range 135-200 nM, the calcium bound states had high ECs (116 mM^{-1} cm^{-1} for RTT and 100 mM^{-1} cm^{-1} for NRS).

Random mutagenesis

We next opened two different evolutionary branches, starting from either NRS or RTT and kept on mutagenizing and screening both parental constructs further using random mutagenesis procedures. In particular, the mutation F241L showed a positive impact in the response at the cost of a small reduction of brightness. This region was also critical for the kinetics, with the mutation of F241Y leading to faster off-kinetics at the expenses of a reduced EC. Interestingly, the side chain of this amino acid is directed towards

the chromophore inside the β-barrel, indicating a role of the internal network of interactions in the chromophore transitions induced for calcium binding. We stablished that the best performances were obtained with RTT F241L and NRS F241Y. At this stage of evolution, we had achieved high responses both with purified proteins (> 7000 %) with extinction coefficients corresponding to 80-90% of the values for the original mNeonGreen protein. However, we detected a reduced expression level of these variants in mammalian cells and bacteria compared to control indicators. We therefore focused the efforts at increasing the expression levels, folding and solubility, while monitoring carefully that the brightness, response and calcium binding affinity remained in the desired range. This was achieved by performing several rounds of random mutagenesis screenings in bacterial plates and further testing of selected variants in mammalian cells. During these steps, around 130000 colonies were evaluated. The general strategy consisted in identifying mutations leading to increases in expression and further evaluation of the impact of these modifications in the performance of the indicator. If an amino acid position showed interesting effects on other parameters besides expression, site saturation mutagenesis and further screenings were performed on those positions. We used different strategies to generate variability by means of PCR: i) Using degenerate primers in only one amino acid position, ii) combining 2-5 primers containing specific identified mutations for different amino acids or iii) including multiple degenerated primers, each one corresponding to one amino acid position. In many cases, we found mutations that led to strong increases in the expression levels without any apparent effect on the properties measured on purified proteins, but completely abolished the response in mammalian cells. One of these cases was the mutation M100V, whose side chain is directed inside the beta barrel. This highlighted the need of evaluating at every step the selected candidates in mammalian cells. Some of the amino acid positions that were individually evaluated by site directed mutagenesis were A11, T37, W61, M100, A146, C159, D198, M215, K229, F241, S255, T246, R258 and M277.

Crystal structure

During the mentioned screenings, we expressed and characterized more than 300 variants, generating a detailed map of the effect that different amino acids had on the indicator performance, and identified several indicators with interesting properties derived from both the NRS and RTT parental variants. We then aimed to obtain a crystal structure that could allow us to rationalize the information obtained during the screenings and further evolve the variants. After several attempts of crystallization, we were successful with one variant (Named NRS 1.2) derived from the NRS F241Y parental construct that contained the extra mutations A11G/T37S/R258I (Fig. 1a, Supplementary Fig. 1). The incorporation of these 3 mutations led to a 10-fold increase in expression levels without deteriorating the response, brightness or Kd of the parental construct. In addition, we removed the last nine C-terminus amino acids (VMGMDELYK) to reduce the presence of flexible regions during the crystallization. The crystal structure (Supplementary Fig. 1) showed many interesting functional features of the indicator. As was also observed in the case of Twitch-2B, a residue of linker 2, that in our case was Leu221, stablish strong interactions with a hydrophobic cluster in TnC

(Supplementary Fig. 1a). In addition, multiple amino acids of both linkers, TnC and mNeonGreen establish polar and hydrophobic interactions in these regions (Supplementary Fig. 1b). Importantly, Leu148 (first amino acid of linker 1) is positioned in close proximity to the chromophore, likely playing an important role in stabilizing the closed conformation of the β-barrel and the anionic form of the chromophore (Supplementary Fig. 1c). Another critical residue pointed by the obtained structure is Asn225. This amino acid (originally a cysteine in mNeonGreen) acts as a bridge between the calcium binding-domain and the mNeonGreen β-barrel structure (Supplementary Fig. 1d) establishing clear polar interactions with both domains. Finally, aligning the indicator with the published structure of mNeonGreen (PDB 5ltr), it becomes evident that the insertion of the calcium binding domain in the β-strand containing amino acid residues 135-145 of mNeonGreen induces a higher degree the flexibility and thus a large conformational change not only in the mentioned strand but also in the region consisting of amino acid residues 190-200 (Supplementary Fig. 1e). Finally, we also observe a significant change in the position of the chromophore (Supplementary Fig. 1f) that might explain the shift in the excitation maximum from 507 to 497 nm characteristic of the NRS variants. It is interesting to mention that most of the mutations increasing solubility, folding and expression were located in mNeonGreen and with a few exceptions, their side chains were pointing outside the β-barrel.

Developing ultra-low affinity variants

At this point, we had obtained different indicators evidencing about 80-90% of the brightness of mNeonGreen in the calcium bound state, a high response in solution and appropriate expression levels. However, the calcium binding affinity was typically in the range of 200 nM, far too high for any interstitial calcium measurements. In this regard, the last step was to test mutations previously described in TnC with the aim of reducing the binding affinity[1]. We found two insertions in the calcium binding EF-hand loops (169D+ and 205S+) being the most effective at drastically reducing the binding affinity with a minor impact in the protein response and brightness. While inserting these residues in the NRS templates led to variants with dissociation constants in the μM range, the incorporation of the same mutations in the RTT derivatives, led to the desired mM range in the calcium binding affinities. We selected a candidate containing the mentioned A11G/T37S/R258I mutations, plus G248D, that also led to higher expression levels without affecting any other parameter. The insertion of 169D and 205S led to the variants GreenT-EC and GreenT-EC.b respectively. Including the additional amino acid exchange T260I, discovered separately, in GreenT-EC.b, further reduced the binding affinity to generate GreenT-EC.c.

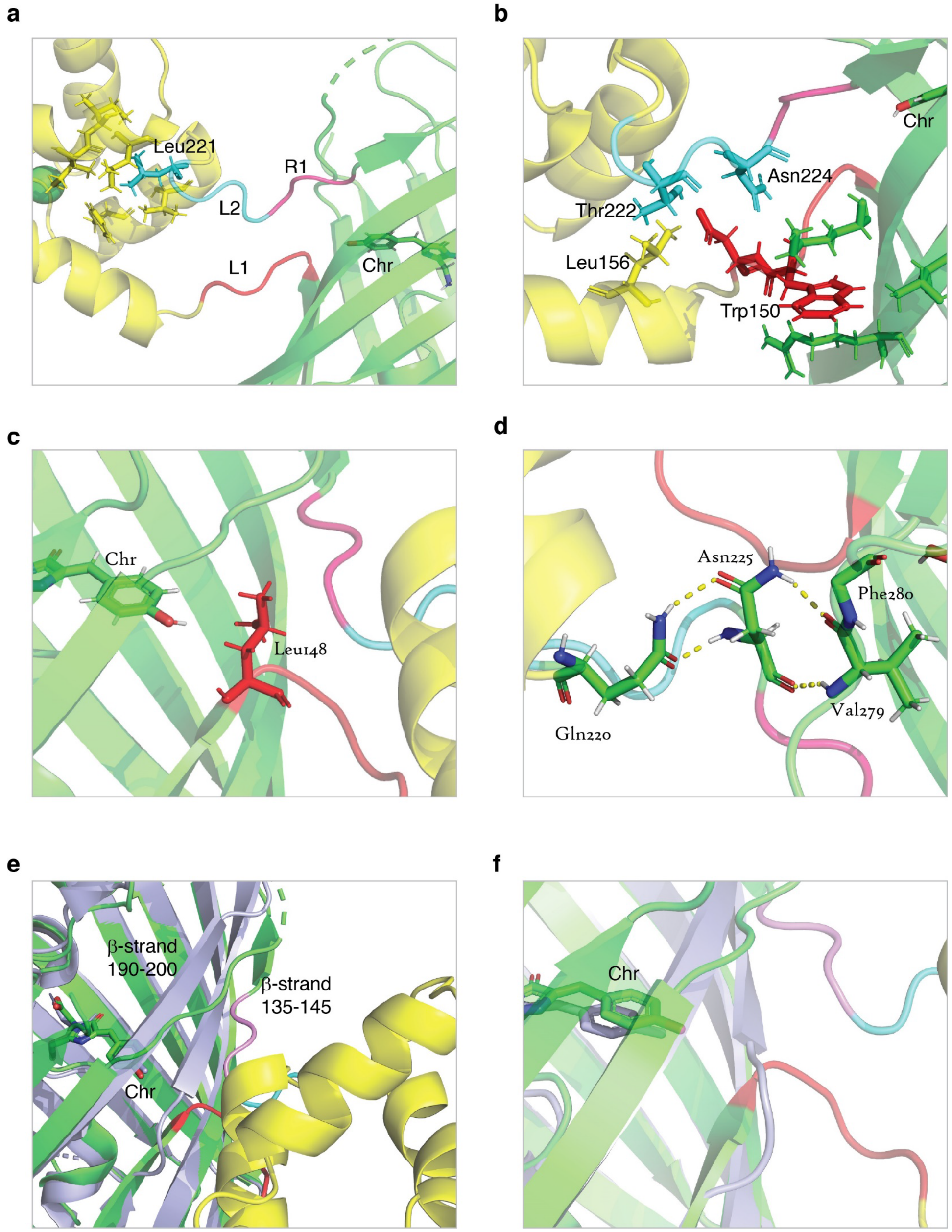

Supplementary Figure 1. Structural details of NRS 1.2. mNeonGreen is depicted in green and TnC domain in yellow. In addition, linker 1 (L1) and linker 2 (L2) are represented in red and cyan respectively. In red it is indicated the region 1 (R1) which correspond to the three amino acids 225-227 of the sensor and are located within the mNeonGreen coding region immediately after linker 2. **a)** Interaction of Leucine 221 present in linker 2 with a hydrophobic patch in TnC. **b)** Multiple interactions involving the linkers and both TnC and mNeonGreen help to stabilize the calcium bound conformation. **c)** Important role of the side chain of leucin 148 in linker 1 facing the chromophore and closing the ß-barrel. **d)** Asparagine (Asn) 225 establishes simultaneous polar interactions with residues in the ß-barrel of mNeonGreen at amino acid residues Val279 and Phe280 and TnC residue Gln220. **e)** and **f)** show an overlay of NRS 1.2 with mNeonGreen (violet) evidencing changes in the conformation of the native mNeonGreen ß-barrel residues 135-145 and 190-200 as well as in the position of the chromophore. Chr indicates the position of the chromophore.

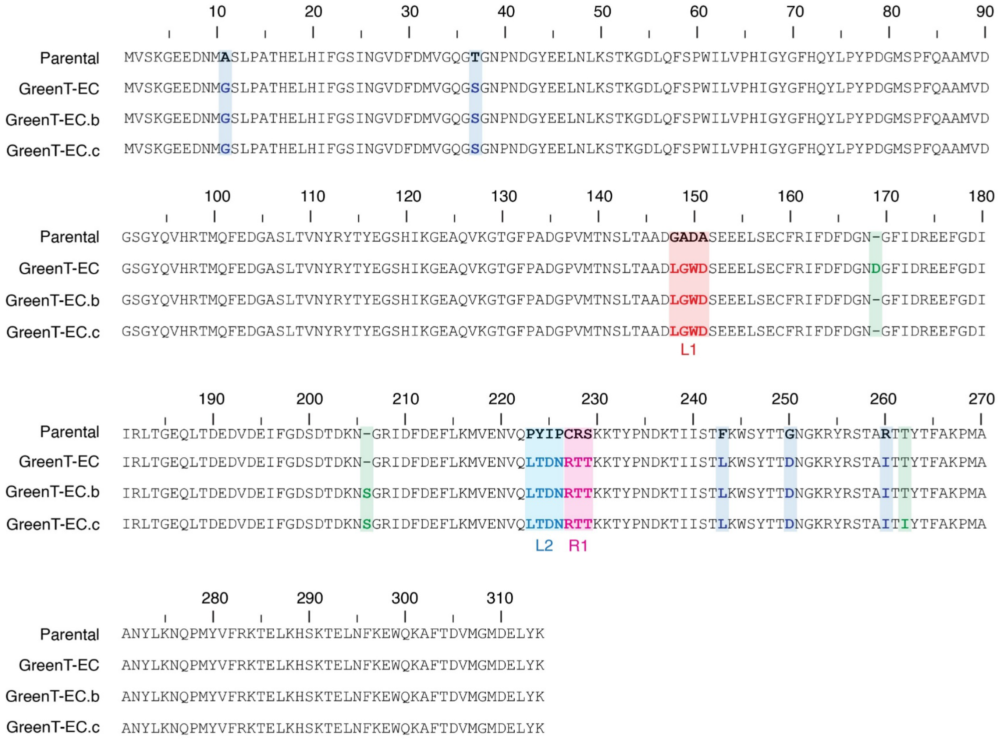

Supplementary Figure 2. Amino acid sequences of GreenT-ECs and the parental protein. The amino acid sequence of the parental protein that served as starting point for directed evolution of GreenT-ECs is shown in the top row. While GreenT-EC contains an insertion of an aspartic acid after residue 168 in the in the first calcium binding EF-hand motif (colored green), GreenT-EC.b and GreenT-EC.c contain insertions of a serine (colored green) after position 204 within the second calcium binding EF-hand motif. GreenT-EC.c contains an additional amino acid exchange (T260I, green). Engineered residues of linker 1 (L1) between mNeonGreen and the minimal TnC domain are in red. Residues marked in blue represent the engineered linker 2 (L2) between mNeonGreen and the minimal calcium binding domain. Residues highlighted in magenta adjacent to the TnC domain correspond to region R1. Amino acid exchanges in mNeonGreen that enhanced response, folding and expression of GreenT-ECs are colored dark blue. Numbering is based on the parental construct. The insertion of D169 or S205 will shift it accordingly.

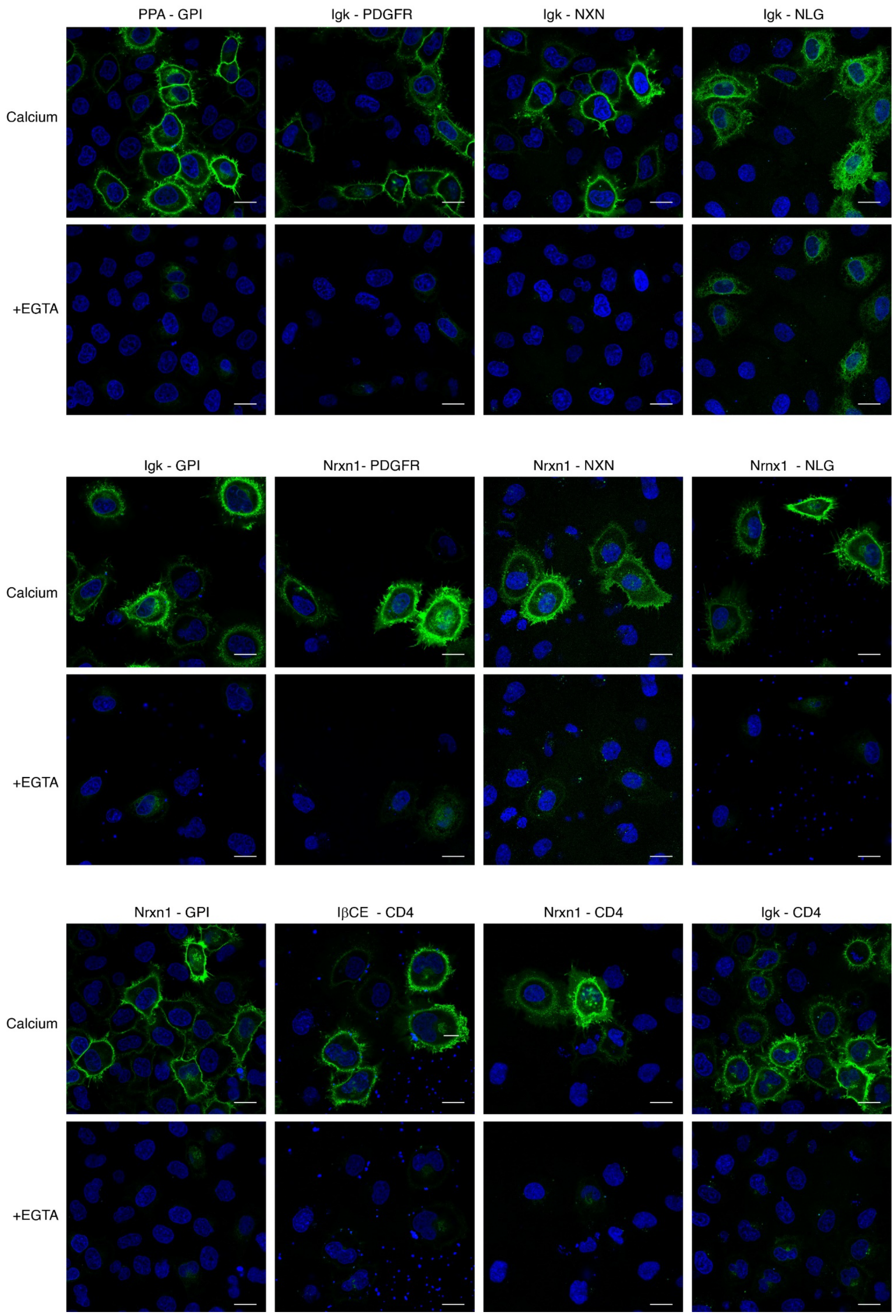

Supplementary Figure 3. Surface delivery of GreenT-ECs with various targeting motifs. Exemplary images show expression of constructs in which different surface targeting sequences were fused to GreenT-EC. Cells are shown with either 3 mM extracellular calcium concentration in HBSS buffer or at zero extracellular calcium using 3 mM EGTA. GreenT-EC fluorescence (green) and Hoechst 33342 nuclear counterstain (blue) are displayed. Scale bar, 20 µm. CD4: transmembrane domain of the T-cell surface glycoprotein Cluster of Differentiation 4. PPA: N-terminal 24 amino acids of mouse preproacrosin signal peptide. PDGFR: Homo sapiens platelet derived growth factor receptor beta; NXN: rat neurexin-1β; NLG: rat neuroligin-1. GPI: mouse Thy-1 glycosylphosphatidylinositol anchoring domain; Igk: N-terminal 21 aa from human CH29 light chain; Nrxn1: N-terminal 63 aa of mouse Neurexin I; IßCE: N-terminal 29 amino acids from Integrin-ß of *C. elegans*.

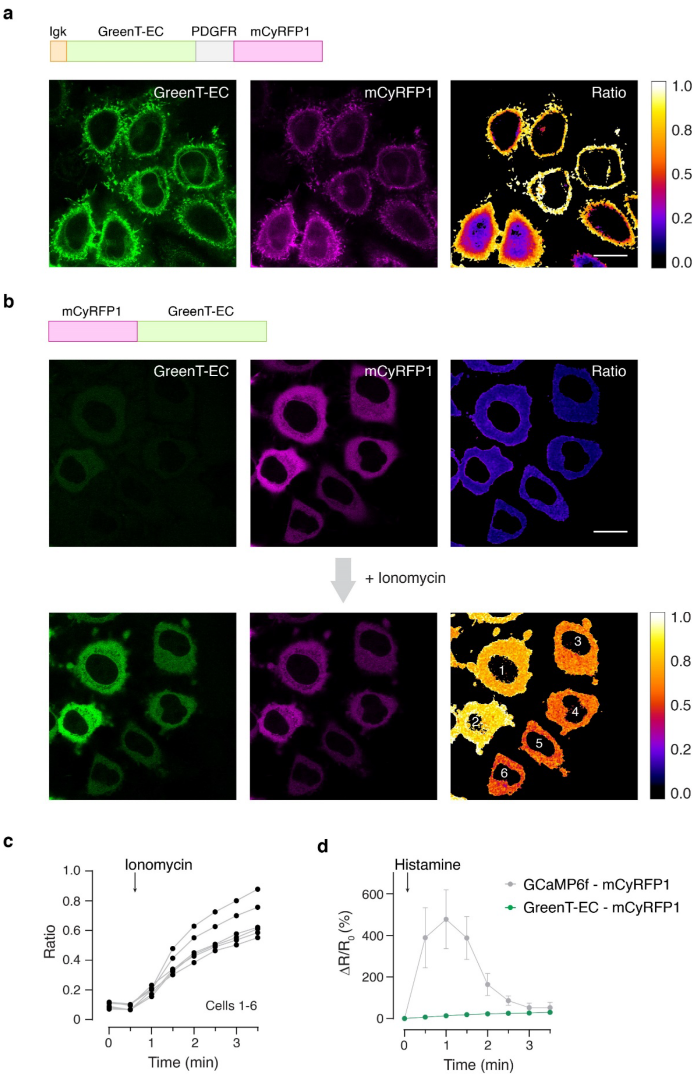

Supplementary Figure 4. Cytosol vs Surface Localization of GreenT-EC in HEK293 cells. a) GreenT-EC fluoresces brightly when cell surface localized and exposed to high calcium extracellular buffer. Green channel: GreenT-EC fluorescence. Red channel: mCyRFP1 reference protein. Ratio: GreenT-EC/mCyRFP1. **b)** GreenT-EC is essentially non-fluorescent when localized within the cytosol and only shows detectable fluorescence upon permabilization of the cells to the extracellular Ca^{2+} concentration (1.44 mM) with Ionomycin 2.5 µM. Scale bar, 50 µm. **c)** Plot of the ratio evolution after the addition of ionomycin of the cells indicated in b. **d)** The response to physiological increases in the intracellular calcium concentrations of cytosolic GreenT-EC (n=30 cells, one transfected dish) was compared to GCaMP6f (n=15 cells, one transfected dish). Bars represent the standard error of the mean. Source data is available in the Data Source file.

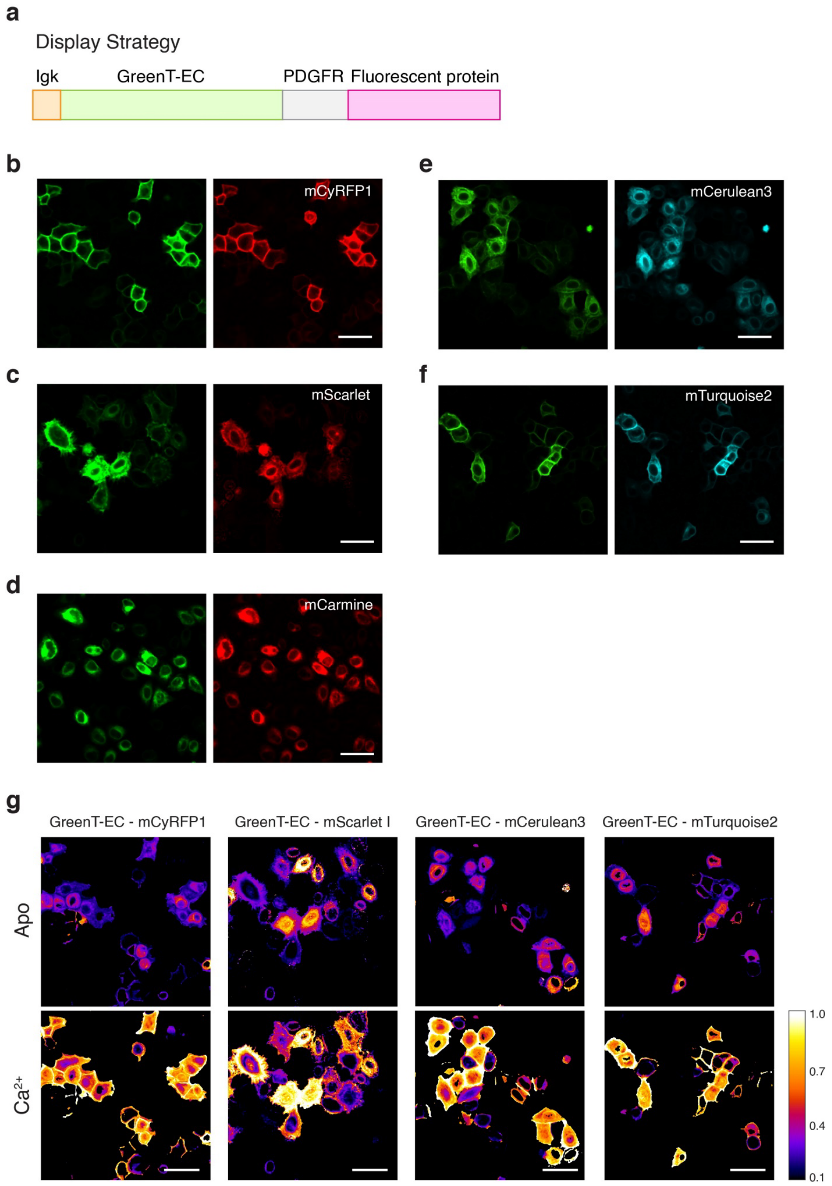

Supplementary Figure 5. Fusions of reference proteins to the cytosolic tail of GreenT-EC. a) Schematic representation of the surface-targeted GreenT-EC construct used for evaluation. It consists of the export signal peptide (Igk), the sensor (GreenT-EC), the transmembrane domain (PDGFR) and the cytosolic fusion of the reference proteins. The use of mCyRFP1 **(b)**, mScarlet-I **(c)**, mCarmine **(d)**, mCerulean3 **(e)** and mTurquoise2 **(f)** was evaluated in HeLa cells in the presence of 3 mM Ca^{2+} and its performance was qualitatively rated in terms of expression and membrane/cytosol contrast (translocation). While mScarlet, mCyRFP1, mCerulean3 and mTurquoise2 showed a clear contrast membrane/cytosol, mCarmine was only visible in the cytosol indicating that is accumulated inside cells during protein translocation. **g)** Ratiometric images GreenT-EC/Reference protein were calculated in the presence of Ca^{2+} 3 mM before and after the addition of EGTA 3 mM. Scale bar, 50 µm.

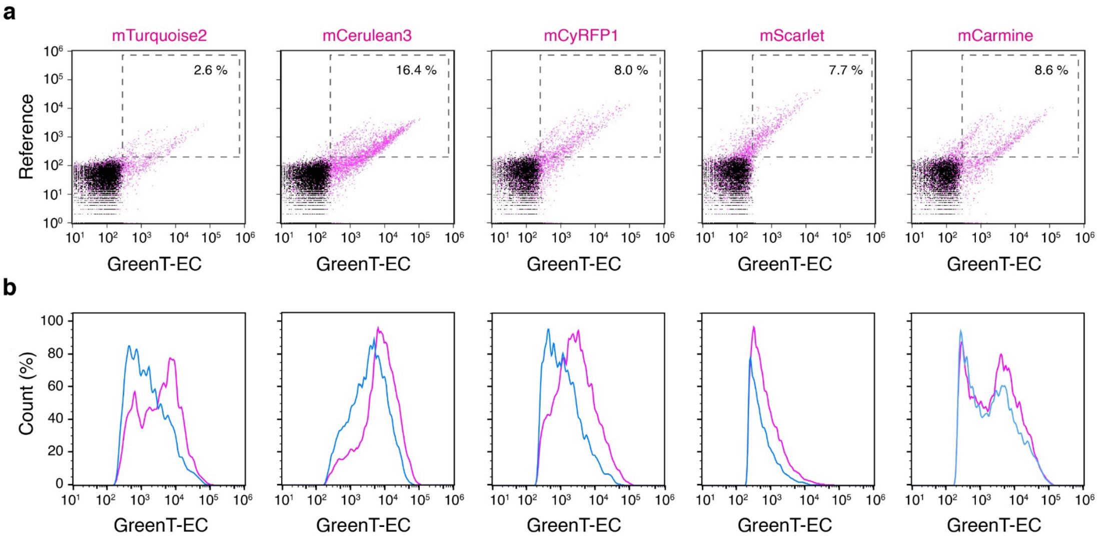

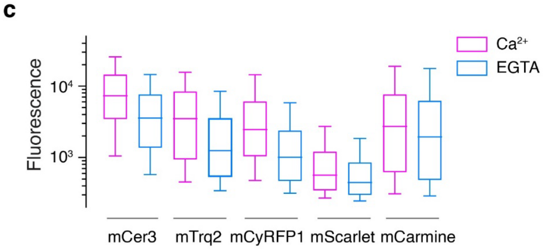

d

Reference protein	Condition	Cell count	Mean fluoresc.	s.e.m.	$\Delta R/R_0$ (%)
mCerulean3	Ca^{2+}	12421	11088	106	86
	EGTA	12395	5963	119	
mTurquoise2	Ca^{2+}	1691	6588	143	85
	EGTA	1593	3569	193	
mCyRFP1	Ca^{2+}	5900	5834	169	129
	EGTA	5774	2547	189	
mScarlet	Ca^{2+}	5998	1492	268	46
	EGTA	4367	1022	249	
mCarmine	Ca^{2+}	6543	7002	169	9
	EGTA	6430	6413	188	

Supplementary Figure 6. Flow cytometry quantification of referenced GreenT-EC cell surface localization. Five different fluorescent proteins were fused at the C-terminus of the construct Igk-GreenT-EC-PDGFR that covered the range of Cyan-yellow-red region of the spectra. HeLa cells were transiently transfected with each construct and further analyzed using flow cytometry. **a)** Overlay density plots of the different constructs and the negative control (non-transfected) indicating the gating strategy for selecting GreenT-EC-Reference positive single cells (slashed lines). Both the negative and the rest of the construct displayed were resuspended in MOPS buffer containing $CaCl_2$ 3 mM. **b)** Histograms of the gated population before and after the addition of EGTA (final concentration 7.5 mM). **c)** Box-plots 90-10% (whiskers are 90 and 10 percentiles, boxes are 75 and 25 percentiles and the center line correspond to the median) of the GreenT-EC fluorescence of gated cells before and after the addition of EGTA. **d)** Summary table compiling the main parameters: Number of cells gated as GreenT-EC positive in each condition (n = Cell count), mean fluorescence of GreenT-EC (Mean fluoresc.), standard error of the mean (s.e.m.) and the response calculated between the mean GreenT-EC fluorescent values before and after the addition of EGTA ($\Delta F/F_0$). Source data is available in the Data Source file.

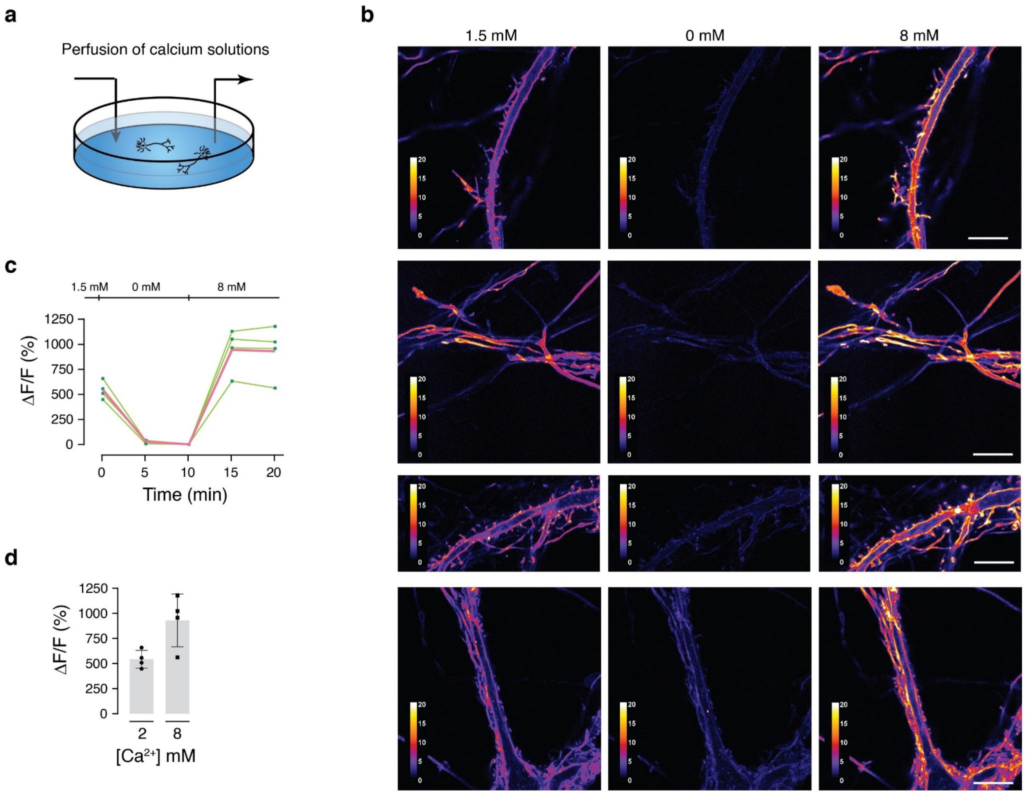

Supplementary Figure 7. GPI-anchored GreenT-EC in primary hippocampal neurons. Confocal time-lapse imaging of GreenT-EC fluorescence intensity changes in response to different extracellular calcium concentrations. a) Schematic representation of perfusion experiments**. b)** Images used to quantify fluorescence changes are displayed using a pseudo-color LUT to facilitate visualization of the extremely dim 0 mM calcium condition. Scale bar, 10 μm. **c)** Quantification of fluorescence changes compared to 0 mM point respectively during the perfusion experiments. Each line represents the results obtained in one dish and the red trace correspond to the mean value of all four. **d)** Bar plot of the responses presented in (c), using the values at 0 mM Ca^{2+} as the minimum fluorescence level. Bar represent the mean value and error bars are standard deviations (n=4 biological replicates). Source data is available in the Data Source file.

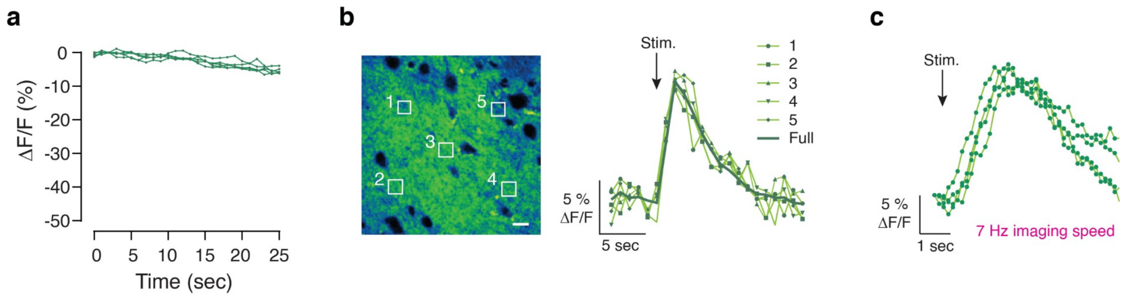

Supplementary Figure 8. Photobleaching of GreenT-EC and further representative GreenT-EC responses. a) Photobleaching effect of GreenT-EC expressing hippocampal organotypic slices (n=5 biological replicates). **b)** Representative example of GreenT-EC expressing hippocampal organotypic slice (left) and the fluorescence traces upon electrical stimulation (right). White ROIs shown in the image correspond to individual traces. As observed in Fig. 5b the responses of cell bodies are homogenous in different regions and accurately averaged when considering the full window (dark-green trace). Scale bar, 10 μm. **c)** GreenT-EC fluorescence responses upon neuronal electrical stimulation with 7 Hz imaging speed (n=4 biological replicates). Source data is available in the Data Source file.

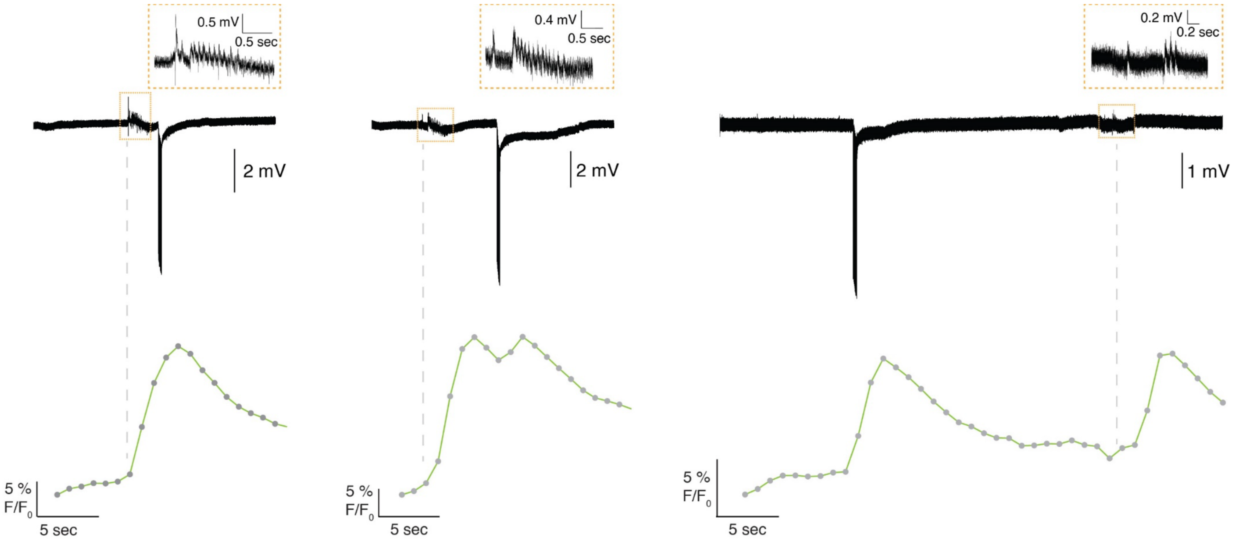

Supplementary Figure 9. Spontaneous activity induces GreenT-EC signals in hippocampal slices. Spontaneous activity was correlated when early (before stimuli) or late (after stimuli) GreenT-EC responses were observed. In the upper panel, the electrophysiology recordings are displayed with a zoomed inset into the spontaneous activity window. In the bottom panel, GreenT-EC fluorescence traces are presented as F/F_0. Source data is available in the Data Source file.

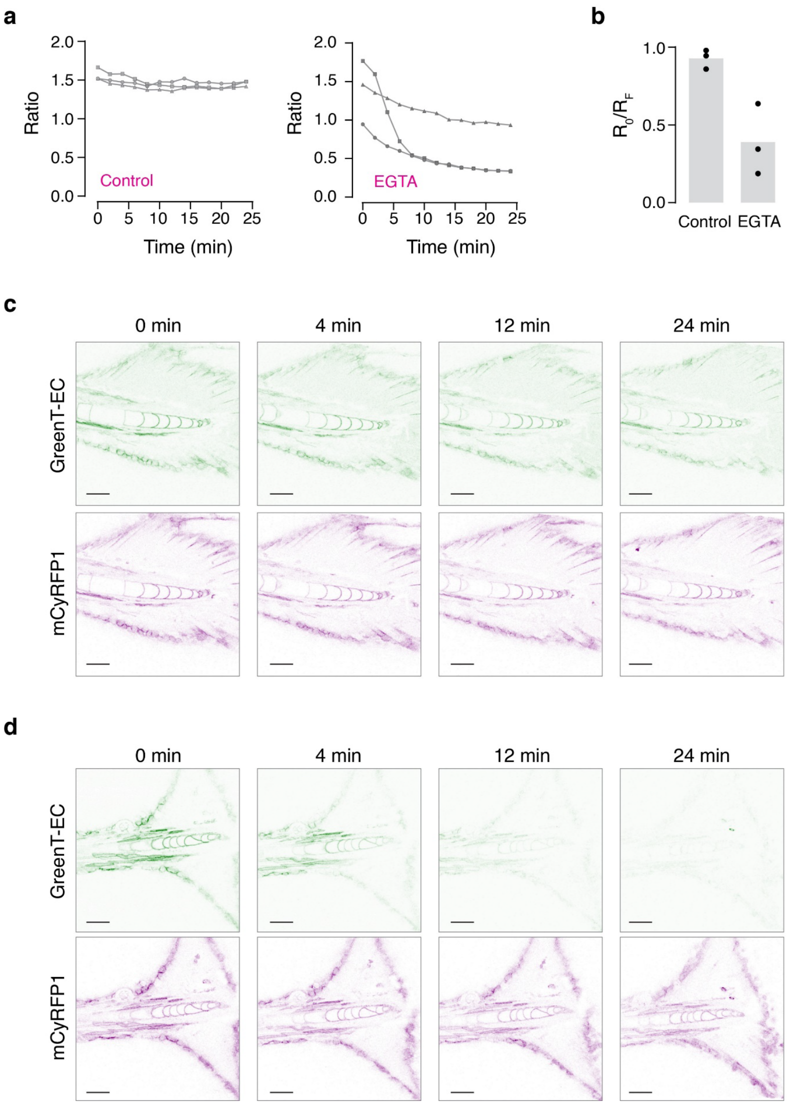

Supplementary Figure 10. Time course of EGTA administration to the fin fold of zebrafish embyos. a) Time-evolution curves of the ratio values for control animals or EGTA-treated during 60 seconds before imaging. Each trace corresponds to one zebrafish embryo. **b)** Quantification of GreenT-EC/mCyRFP1 ratios in control (n= 3 biological replicates) and 5 mM EGTA treated animals (n=3 biological replicates) at the end of the time-lapsed experiment. **c-d)** Representative confocal images from time-lapse imaging animals of the control or 5 mM EGTA-treated groups, respectively. Scale bar, 50 μm. Source data is available in the Data Source file.

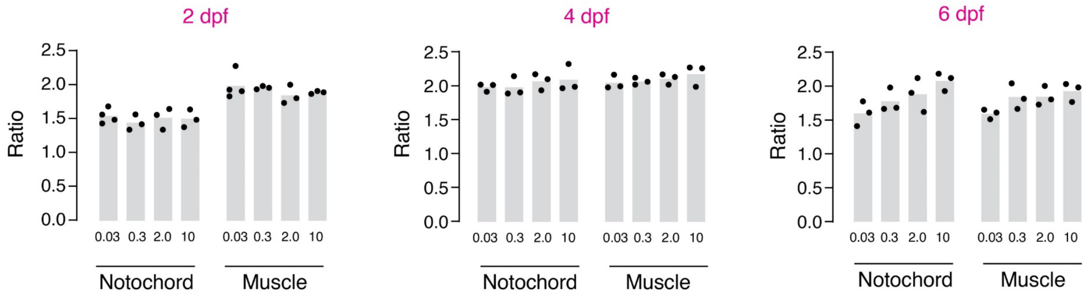

Supplementary Figure 11. Effects of large concentration differences of environmental calcium on interstitial calcium in fish larvae. Fish were raised to 2, 4 or 6 dpf (days post fertilization) in E2 embryo medium containing from 0.03 mM to 10 mM calcium and the effects on interstitial calcium were quantified in notochord and muscle tissue. For all experiments each dot represents the mean of ≥ 6 cells for one fish. For each condition 3 animals (n=3) were analyzed. Only for the case of 0.03 mM Ca^{2+} at 2 dpf, four animals were evaluated (n=4). Source data is available in the Data Source file.

Supplementary References

1. Thestrup, T. et al. Optimized ratiometric calcium sensors for functional in vivo imaging of neurons and T lymphocytes. *Nat Methods* **11**, 175-182 (2014).
2. Trigo-Mourino, P., Thestrup, T., Griesbeck, O., Griesinger, C. & Becker, S. Dynamic tuning of FRET in a green fluorescent protein biosensor. *Sci Adv* **5**, eaaw4988 (2019).
