# Supplementary material for: Fluorescent sensors for imaging of interstitial calcium
Source: Nat Commun. 2023 Oct 5;14:6220. doi: 10.1038/s41467-023-41928-w (PMC10556026; doi:10.1038/s41467-023-41928-w)
Supplement: Supplementary file 3 — Reporting Summary [file 41467_2023_41928_MOESM3_ESM.pdf]

nature portfolio

Corresponding author(s): Oliver Griesbeck

Last updated by author(s): Sep 18, 2023

Reporting Summary

Nature Portfolio wishes to improve the reproducibility of the work that we publish. This form provides structure for consistency and transparency in reporting. For further information on Nature Portfolio policies, see our Editorial Policies and the Editorial Policy Checklist.

Statistics

For all statistical analyses, confirm that the following items are present in the figure legend, table legend, main text, or Methods section.

n/a	Confirmed	
☐	☒	The exact sample size (*n*) for each experimental group/condition, given as a discrete number and unit of measurement
☐	☒	A statement on whether measurements were taken from distinct samples or whether the same sample was measured repeatedly
☐	☒	The statistical test(s) used AND whether they are one- or two-sided<br>*Only common tests should be described solely by name; describe more complex techniques in the Methods section.*
☒	☐	A description of all covariates tested
☐	☒	A description of any assumptions or corrections, such as tests of normality and adjustment for multiple comparisons
☐	☒	A full description of the statistical parameters including central tendency (e.g. means) or other basic estimates (e.g. regression coefficient) AND variation (e.g. standard deviation) or associated estimates of uncertainty (e.g. confidence intervals)
☐	☒	For null hypothesis testing, the test statistic (e.g. *F*, *t*, *r*) with confidence intervals, effect sizes, degrees of freedom and *P* value noted<br>*Give P values as exact values whenever suitable.*
☒	☐	For Bayesian analysis, information on the choice of priors and Markov chain Monte Carlo settings
☒	☐	For hierarchical and complex designs, identification of the appropriate level for tests and full reporting of outcomes
☒	☐	Estimates of effect sizes (e.g. Cohen's *d*, Pearson's *r*), indicating how they were calculated

Our web collection on statistics for biologists contains articles on many of the points above.

Software and code

Policy information about availability of computer code

Data collection	We used ImageJ macro routines to process the images and measure the mean pixel values. We used Python routines for acquisition and analysis of bacterial plates during the screening stage of the project, previously developed by our group (Fabritius et. al. 2018 - Cell Chem Biol 25, 1554-1561 e1558). All ImageJ macros and Python scripts can be found in the Source Data file and in Zenodo (https://doi.org/10.5281/zenodo.8355596).
Data analysis	Data was plotted and analyzed using Graphpad Prism.

For manuscripts utilizing custom algorithms or software that are central to the research but not yet described in published literature, software must be made available to editors and reviewers. We strongly encourage code deposition in a community repository (e.g. GitHub). See the Nature Portfolio guidelines for submitting code & software for further information.

Data

Policy information about availability of data

All manuscripts must include a data availability statement. This statement should provide the following information, where applicable:

- Accession codes, unique identifiers, or web links for publicly available datasets
- A description of any restrictions on data availability
- For clinical datasets or third party data, please ensure that the statement adheres to our policy

All data generated in this article can be found in the Source Data file. Mammalian and bacterial expression vectors coding for GreenT-ECs are available from Addgene (Accession Nos. 193943 to 193949). Crystal structure: The coordinates and the structure factors have been deposited in the Protein Data Bank (PDB) under

the accession number: 8C0T. Source data is provided with this article.

Human research participants

Policy information about studies involving human research participants and Sex and Gender in Research.

Reporting on sex and gender	N/A
Population characteristics	N/A
Recruitment	N/A
Ethics oversight	N/A

Note that full information on the approval of the study protocol must also be provided in the manuscript.

Field-specific reporting

Please select the one below that is the best fit for your research. If you are not sure, read the appropriate sections before making your selection.

[x] Life sciences [ ] Behavioural & social sciences [ ] Ecological, evolutionary & environmental sciences

For a reference copy of the document with all sections, see nature.com/documents/nr-reporting-summary-flat.pdf

Life sciences study design

All studies must disclose on these points even when the disclosure is negative.

Sample size	No sample size calculation were made. We determined a minimum sample size based on reported standards in the field and our own experience of animal-to animal, cellular and protein batch productions variability. At least three biological samples were analyzed for all cellular and in vivo studies.
Data exclusions	No data were excluded
Replication	Along the studies, the experiments were validated by adding a control sample every time in order to compare the results among different days, months and years. Depending on the particular parameter there were multiple instances of measurements. For instance, in vitro response of the indicators is measured every time the protein is purified. The Kd titration in the cell surface were done in different buffers, days and using referenced and non-referenced constructs, with the data being very reliable. Zebrafish control values were reproduced by at least two experimenters over a spam of three years. In the case of brain slices, control stimulations and treatments they were reproduced also using different virus batches over at least two years.
Randomization	The zebrafish animals for the study were selected and allocated in groups randomly. For brain slices, the comparison is made before and after treatment so no sample allocation into groups was done.
Blinding	No participant groups are involved in this work. Blinding was not adopted during sample allocation and data analysis was performed objectively using macro routines following standard strategies for image processing.

Reporting for specific materials, systems and methods

We require information from authors about some types of materials, experimental systems and methods used in many studies. Here, indicate whether each material, system or method listed is relevant to your study. If you are not sure if a list item applies to your research, read the appropriate section before selecting a response.

Materials & experimental systems

n/a	Involved in the study	
x		Antibodies
	x	Eukaryotic cell lines
x		Palaeontology and archaeology
	x	Animals and other organisms
x		Clinical data
x		Dual use research of concern

Methods

n/a	Involved in the study	
x		ChIP-seq
	x	Flow cytometry
x		MRI-based neuroimaging

Eukaryotic cell lines

Policy information about cell lines and Sex and Gender in Research

Cell line source(s)	HEK 293T - ATCC CRL-3216 / HELA - DSMZ ACC 57
Authentication	Cell lines were not authenticated
Mycoplasma contamination	Cell lines were tested for mycoplasma and a negative result was obtained
Commonly misidentified lines (See ICLAC register)	None

Animals and other research organisms

Policy information about studies involving animals; ARRIVE guidelines recommended for reporting animal research, and Sex and Gender in Research

Laboratory animals	18 day pregnant embryonic Sprague-Dawley rats were used to prepare hippocampal neurons. P5-7 old C57Bl/6J mouse pups were used to obtain hippocampal slices. Zebrafish lines were derived from AB strain, and 1 - 6 dpf animals were used depending on the experiment (indicated in the legend and methods of the manuscript).
Wild animals	No wild animals were used in this study.
Reporting on sex	Sex was not identified as a significant biological variable for our study. Regarding the mice experiments, we did not distinguish between males and females among the perinatal pups used for organotypic cultures, as potential anatomical and/or physiological differences between the two sexes were considered irrelevant in the context of this study. In zebrafish as well, animals of all groups were selected randomly.
Field-collected samples	No field-collected samples were used in this study.
Ethics oversight	Mouse/rat experiments: European directive on the protection of animals used for scientific purposes (2010/63/EU). Zebrafish: For zebrafis studies: experiments with all transgenic lines were performed under standard conditions as per the Federation of European Laboratory Animal Science Associations (FELASA) guidelines, and in accordance with institutional (Université Libre de Bruxelles (ULB)) and national ethical and animal welfare guidelines and regulation, which were approved by the ethical committee for animal welfare (CEBEA) from the Université Libre de Bruxelles (protocols 578N-579N).

Note that full information on the approval of the study protocol must also be provided in the manuscript.

Flow Cytometry

Plots

Confirm that:

- [x] The axis labels state the marker and fluorochrome used (e.g. CD4-FITC).
- [x] The axis scales are clearly visible. Include numbers along axes only for bottom left plot of group (a 'group' is an analysis of identical markers).
- [x] All plots are contour plots with outliers or pseudocolor plots.
- [x] A numerical value for number of cells or percentage (with statistics) is provided.

Methodology

Sample preparation	HeLa cells were seeded in 35 mm dishes (Corning) and transfected with the different constructs according to the manufacturer instructions (Lipofectamine 3000). Twenty-four hours after transfection, cells were detached with Versene solution (Gibco, ThermoFisher) and collected in HBSS supplemented with CaCl2 3 mM to inactivate the detachment agent. After centrifugation at 1000 g during 5 min, cells were resuspended in 1.5 ml MOPS buffer supplemented with 3 mM CaCl2 / 1 mM MgCl2, and transferred to 5 ml round bottom tubes with cell strainer snap cap (Corning).
Instrument	Attune NxT Analyzer cytometer (ThermoFisher Scientific)
Software	FlowJo v10.8.1
Cell population abundance	Sorting was not performed. Analysis of transfection (GreenT-EC gated positive) cells was reported.
Gating strategy	Events were first gated by forward (FSC) and side (SSC) scattering for selecting cells and excluding doublets as shown in the figures. GreenT-Ec positive cells were gated using SSC as an additional dimension. The gating strategy is indicated in the main Figure 3.

- [x] Tick this box to confirm that a figure exemplifying the gating strategy is provided in the Supplementary Information.
