# Supplementary material for: Fluorescent sensors for imaging of interstitial calcium
Source: Nat Commun. 2023 Oct 5;14:6220. doi: 10.1038/s41467-023-41928-w (PMC10556026; doi:10.1038/s41467-023-41928-w)
Supplement: Supplementary file 4 — Description of Additional Supplementary Files [file 41467_2023_41928_MOESM4_ESM.pdf]

**Description of Additional Supplementary Files**

Supplementary Data 1
Description: Resource table

Supplementary Movie 1
Description: GreenT-EC signals in hippocampal slices during puff application of high calcium (8 mM)
